# Design and Implementation of HD Mapping, Vehicle Control, and V2I Communication for Robo-Taxi Services

**DOI:** 10.3390/s22187049

**Published:** 2022-09-17

**Authors:** Jun Yong Yoon, Jinseop Jeong, Woosuk Sung

**Affiliations:** School of Mechanical System and Automotive Engineering, Chosun University, Gwangju 61452, Korea

**Keywords:** autonomous driving, HD map, disturbance observer, V2I communication

## Abstract

This paper presents our autonomous driving (AD) software stack, developed to complete the main mission of the contest we entered. The main mission can be simply described as a robo-taxi service on public roads, to transport passengers to their destination autonomously. Among the key competencies required for the main mission, this paper focused on high-definition mapping, vehicle control, and vehicle-to-infrastructure (V2I) communication. V2I communication refers to the task of wireless data exchange between a roadside unit and vehicles. With the data being captured and shared, rich, timely, and non-line-of-sight-aware traffic information can be utilized for a wide range of AD applications. In the contest, V2I communication was applied for a robo-taxi service, and also for traffic light recognition. A V2I communication-enabled traffic light recognizer was developed, to achieve a nearly perfect recognition rate, and a robo-taxi service handler was developed, to perform the main mission of the contest.

## 1. Introduction

The robo-taxi is drawing attention, as one of the most rapidly adopted services using autonomous vehicles. Although the introduction of robo-taxis is not as rapid as expected, large-scale tests are currently in progress—notably in the United States [1,2] and in China [3,4,5]—some of which are open to the public, and in revenue service [6,7,8,9].

The main mission of the contest that we entered—the 2020 University Student Autonomous Driving Contest—was to transport passengers to their destination autonomously; in short: robo-taxi services on public roads. In line with how the robo-taxi is expected to serve the public in the future, vehicles in the contest were expected to transport passengers between locations of their choice. The contest did not feature actual passengers hailing from the street; their ride-hailing was instead simulated by tens of virtual calls for a ride, broadcast over a vehicular communication system. In the contest, the vehicular communication system was realized based on vehicle-to-infrastructure (V2I) technology, in which the vehicles and roadside units (RSUs) served as communicating nodes, providing each other with information for the robo-taxi service. Each call primarily consisted of the pick-up and drop-off locations of the virtual passengers. The two-dimensional (2D) distance between these locations was also provided by the call. The distance was factored into points at which the vehicles could win by completing the mission. The points varied according to the mission’s degree of difficulty. More points were awarded if the vehicles completed more challenging missions that involved left turns, underpasses, roads missing from the map, and lanes closed for construction: except for the lanes closed for construction, these scenarios were considered to be static; the lane closed for construction, however, was dynamic, and therefore the location of the lane closure was provided not by the map but by the call. This is one of the actual uses of V2I communication, which is often referred to as a roadwork warning. In consideration of all these factors, the vehicles were required to pick up one of the available calls, and then leave for the pick-up location. None of the answered calls were allowed to be selected again, and the calls being responded to by other vehicles could not be selected either. As the contest was held on public roads, the vehicles were required to drive in accordance with the relevant traffic rules. For safety purposes, traffic in and out of the contest site was restricted to 15 competing vehicles and several safety vehicles from the contest organizer. The safety vehicles were deployed to mimic actual traffic conditions, while supervising the competition at the site. If the vehicles violated traffic rules, earned points were deducted. Ten times more points were deducted if the violated traffic rule was a traffic signal, which was considered more stringent than any other rules. Earned points were also deducted if the vehicles issued a take-over request (TOR) to attendants, to evade unsafe situations. All the factors used to calculate the points are listed with their corresponding weights (see Table 1). Accordingly, the vehicles are ranked according to the points that they accumulated during the contest, which lasted for 1.5 h.

The following core competencies were required for vehicles in the contest to demonstrate the robo-taxi service: mapping; localization [10,11,12,13,14,15,16]; path-planning [17,18]; vehicle control [19,20,21,22,23,24]; and perception [25,26,27,28].

A kind of map needed to be created, often called a high-definition (HD) map, which differs from conventional maps made for human drivers. The HD map created for autonomous driving (AD) incorporates two different types of maps: a pointcloud map and a vector map. These two maps commonly represent the geographical features required for AD. Whereas the pointcloud map represents the three-dimensional (3D) shape of objects in driving environments, the vector map represents their global position. The concerned objects include road and roadside infrastructure, such as lane lines, stop lines, road boundaries, traffic signs, traffic lights, etc. The local pointcloud map is registered, as opposed to the global vector map, generating the HD map. Therefore, the registered pointcloud map can deliver a faithful representation of driving environments, with laser-sharp precision.

Localization refers to the task of determining the ego-vehicle’s current position and orientation within the global frame. The registered pointcloud map can help the vehicle in the localization. Prior map-based localization is performed by matching the scanned pointcloud to the registered pointcloud in the prior map, which enables a positioning error within several centimeters.

Path-planning can be regarded as an extension of localization, in that it requires the current position of the ego-vehicle and the position of the goal location on the global frame. The vector map can aid the vehicle in the path-planning. Global planning refers to the task of finding a path towards the goal location. Static obstacles positioned within the vector map are used as prior information to render the path optimal. The obstacles widely denote driving environments that can increase the cost of traversal. For instance, when finding the optimal path, left turns, underpasses, and roads off the map are not desired, because of their higher cost. Local planning refers to the task of generating local trajectories based on the global path. Indeed, the best trajectory must be drivable by the ego-vehicle and collision-free from dynamic obstacles. The local planner is integrated with the behavior planner, which leads the vehicle to the goal location safely and efficiently.

The planned path is composed of an array of waypoints. Vehicle control enables the ego-vehicle to follow the waypoints given by path-planning. The waypoint follower generates a set of linear and angular speed references. The subsequent speed controller generates a set of throttle, brake, and steering commands, to achieve the referenced speeds that lead the vehicle to the next target waypoint within the next control cycle.

Last but not least, perception refers to the task of extracting relevant information from driving environments to make a decision as to what to do next. Among the core competencies required for the robo-taxi service, perception—which incorporates localization, recognition, and V2I communication—must be the most computationally intensive.

**Figure 1:**
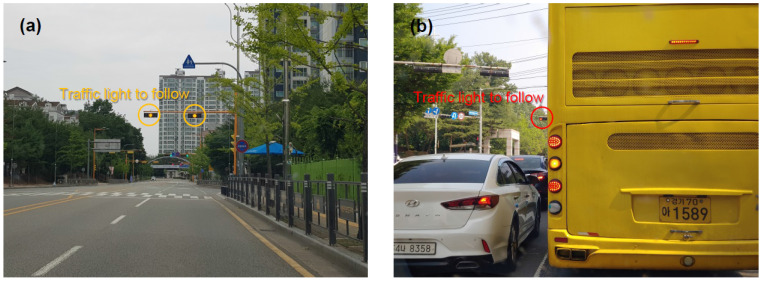
Limitations of a vision-based TLR (**a**) at adjacent intersections, and (**b**) right behind high vehicles.

Among the objects that need to be recognized in driving environments, traffic lights are considered the most crucial for the vehicle to avoid collisions at a signalized intersection. Therefore, a traffic light recognizer (TLR) must achieve an almost perfect recognition rate. However, traffic lights are not easy to recognize, when only using a camera, because of practical limitations [29,30]. For instance, when approaching adjacent intersections, if multiple signal heads are detected simultaneously, it is difficult to decide which traffic light to follow (see Figure 1a). The vector map can also be of assistance to the vehicle, for traffic light recognition. As noted above, the vector map represents the global position of the road infrastructure, including traffic lights. Using a robot operating system (ROS) package, named ‘tf’, the current position of an onboard camera can be determined while localizing the ego-vehicle. The tf keeps track of frames used in AD that change over time, such that it can update the current position of the baselink frame relative to the map frame, and that of the camera frame relative to the baselink frame. The current position of the vehicle is determined based on the baselink frame, the origin of which is located at the center of the rear axle. Accordingly, the 3D distance between the signal head and the onboard camera can be estimated and projected onto an image plane. Therefore, the TLR need not scan the entire region of the image plane; instead, it only scans the region-of-interest where the next traffic light is expected to appear. Despite this supplementary measure to improve the recognition rate, making it perfect is still elusive for the vision-based TLR, because of practical limitations. For instance, when approaching an intersection, it is impossible to recognize the next traffic light, if a signal head is occluded from the camera by high vehicles immediately ahead of the ego-vehicle (see Figure 1b).

Currently, a V2I communication-enabled TLR is gaining attention as a definitive measure, because of its non-line-of-sight (NLOS) awareness. V2I communication is the task of wireless-exchanging traffic information between the RSU and vehicles, eventually making AD safer and more efficient. For example, the real-time signal phase and the timing status of the corresponding traffic lights are broadcast by the RSU; V2I communication can help the vehicle, in regard to traffic light recognition and behavior-planning, by providing it with the remaining time of the current phase. Owing to its wide range of applications, V2I communication was employed for traffic light recognition and also for robo-taxi services in the contest.

**Figure 2:**
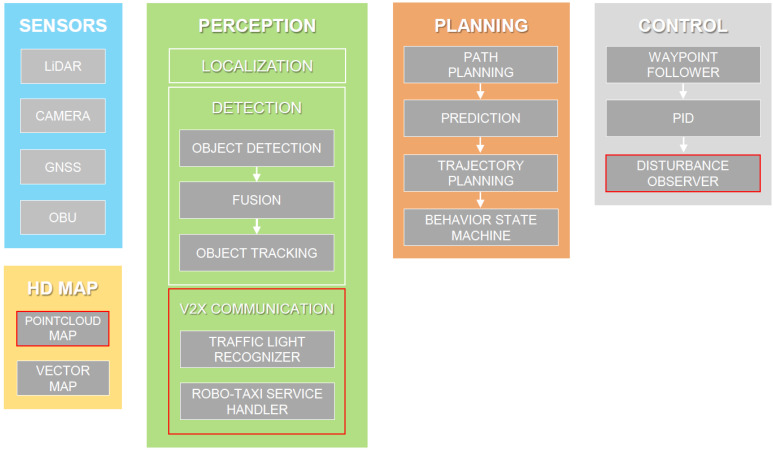
AD software stack developed to win the contest. The major developments are highlighted in red.

In this paper, we introduce our AD software stack, developed to complete the main mission of the contest (see Figure 2). We utilized an open-source AD software platform, Autoware, for rapid prototyping. Autoware can be described as a collection of ROS packages in localization, perception, and path-planning modules [31,32,33]. Among the core competencies required for the robo-taxi service, most of our efforts were centered around the modules that Autoware does not support. Accordingly, this paper centers on HD mapping, vehicle control, and V2I communication. The main potential contributions of this paper are providing practical procedures for developing an HD map, longitudinal speed controller, and the services implemented by V2I technology within the contest. The developed procedures will be beneficial to all concerned, particularly to participants in upcoming autonomous driving events all around the world. The hardware to implement the developed AD stack is summarized in Table 2.

The remainder of this paper is organized as follows. In Section 2, we present the development of an HD map for use in prior map-based localization. In Section 3, we present the development of a disturbance observer (DOB)-based longitudinal speed controller, that is particularly robust against undesired torque variations at low speeds. In Section 4, we present the development of a V2I communication-enabled TLR, to achieve a nearly perfect recognition rate. We also present the development of a robo-taxi service handler (TSH), to perform the main mission of the contest. Finally, we conclude this paper in Section 5.

## 2. HD Mapping

Localization refers to the task of determining the ego-vehicle’s current position, relative to the global frame. AD begins with a localizer. Precise localization is a prerequisite of safe and reliable navigation, particularly in urban environments, where the contest is held. Despite the lack of established standards, it has been reported that the required positioning error is at decimeter-level on highways [34]. On local streets, their road geometry makes the required positioning error more stringent, such that it is down to near-centimeter-level. In addition to road geometry, such as lane width and road curvature, vehicle dimensions also need to be taken into account: this is because such positioning error levels are derived to ensure that the vehicle remains within a lane during AD.

A near-centimeter-level positioning error can be achieved by employing a real-time kinematic global navigation satellite system (RTK-GNSS). However, a GNSS localizer requires a clear line-of-sight to the satellites. Therefore, actual positioning error levels are susceptible to urban environments, especially in long tunnels or near tall buildings. We instead used prior map-based localization, to achieve precise localization even in urban environments [35]. Prior map-based localization, also known as a LiDAR localizer, is achieved by matching the scanned pointcloud against the pointcloud in the prior map. In the map-matching process, the small pointcloud generated by an onboard LiDAR roams about the large pointcloud in the prior map. A certain position that gives the highest level of matching between these two pointclouds gives the current position of the onboard LiDAR and, in turn, that of the vehicle, relative to the map. The map should be relative to the global frame, for determining the global position of the vehicle.

In this section, we introduce a procedure for map-building and subsequent map-matching, to validate the created HD map. The following procedure provides a step-by-step guide on how to transform raw pointcloud data into an HD map, for use in prior map-based localization. We also discuss practical issues that occurred during the procedure (see Table 3).

### 2.1. Map-Building

The map-building procedure comprises laser-scanning, mapping, registration, validation, and annotation steps. The last step can be left out for localization, which would be more significant to path-planning.

#### 2.1.1. Laser-Scanning

**Figure 3:**
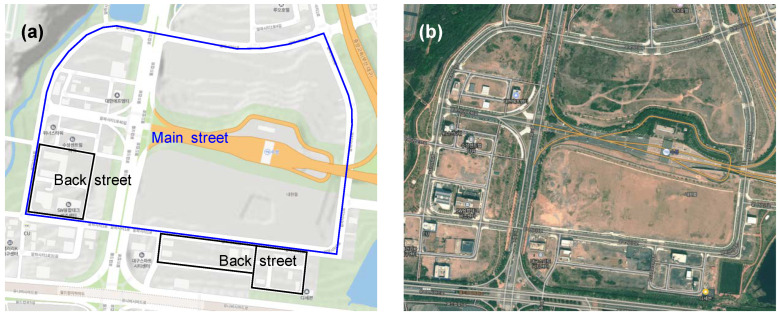
Contest site on the (**a**) topographic and (**b**) satellite maps.

We began map-building by laser-scanning the 3D shapes of objects in driving environments. The vehicle was driven smoothly and consistently around the contest site, preferably in a closed loop. Rather than using dedicated mobile mapping systems (MMS), a 64-channel LiDAR (OS1-64, Ouster), fixed on the roof of the vehicle, was used to collect pointcloud data. The contest site consisted of a main street and several back streets (see Figure 3). While the contest site was laser-scanned in a closed loop, the scanned pointcloud was rosbag-recorded. The rosbag is a logging format for storing ROS messages in files.

#### 2.1.2. Mapping

Subsequently, we selected a mapping package, and ran it on the scanned pointcloud. Autoware provides two different types of ROS packages for map-building: NDT_mapping and approximate_NDT_mapping. As their names imply, both packages are based on normal distributions transform (NDT), which can be also implemented for map-matching. NDT is an algorithm that transforms a raw pointcloud into a grid-based representation [36,37,38,39,40]. The pointcloud in each grid cell is approximated with a multivariate normal distribution. The normal distribution provides a smooth piecewise representation of the pointcloud, with continuous first- and second-order derivatives. Using this representation, it is possible to apply numerical optimization methods for map registration. As a registration algorithm, NDT is fundamentally used to match one pointcloud to another, to organize the map. In contrast to approximate_NDT_mapping, NDT_mapping matches the last pointcloud against all previously matched pointclouds, producing a more precise and larger single map; the mapping process, however, is computationally costly and occasionally even unstable. According to the corresponding ROS specification, even if the mapping process is complete, a single map larger than a certain size (1 GB) cannot be published. As the mapping area—the contest site—was extensive, approximate_NDT_mapping was selected, which yielded smaller, yet less precise, multiple maps. The following issues, A and B, occurred in Step 2, and were solved in Step 3.

A. Pointclouds scanned relative to the local frame.

The current pointcloud map was based on the local frame, the origin of which was located at the point where the onboard LiDAR started scanning. The local frame had to be transformed into the global frame because the pointcloud map for use in prior map-based localization should be relative to the global frame. The global frame generally uses the universal transverse mercator (UTM) system, which provides coordinates on a worldwide flat grid, to ease the calculation of geographical positions. The UTM coordinates divide the world into 60 north–south zones of 6° longitude wide. The origin of each UTM zone lies at the intersection of the equator and the central meridian of the zone. For instance, the UTM zone we are in is called UTM52N, which is between 126° and 132° east, and between the equator and 84° north in the Northern Hemisphere.

B. Pointclouds ill-matched by approximate_NDT_mapping package.

**Figure 4:**
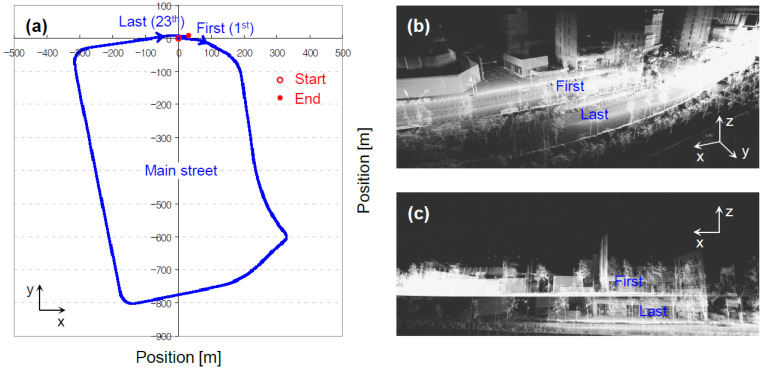
Loose ends between the first and last pointclouds, which are represented (**a**) by line in x–y coordinates, with pointcloud (**b**) in x–y–z coordinates, and (**c**) in x–z coordinates.

The matched pointcloud map was not sufficiently precise, which was revealed by loose ends between the first and last pointclouds (see Figure 4). Their ends needed to be tied up, because the laser-scanning ended exactly where it started. A re-mapping process was therefore required between each of the neighboring pointclouds.

#### 2.1.3. Registration (Re-mapping)

We then registered multiple local pointcloud maps into a single global pointcloud map, on the basis of the vector map. The vector map of the contest site was provided with respect to the origin of the UTM52N zone. Among a variety of road and roadside infrastructure represented by the vector map, road marks primarily including lane lines and stop lines were utilized in the re-mapping process. Such road marks in each pointcloud map were manually registered with those in the vector map. This could be achieved because the laser-scanned road marks in the pointcloud map were visible. In Step 2, approximate_NDT_mapping released multiple individual pointcloud maps, which facilitated stitching them together piece-by-piece, on the basis of the vector map. In doing so, issues A and B could be solved simultaneously. We used CloudCompare (CC) as a tool for manual registration. As the vector map was originally SHP-formatted, it had to be converted to TXT-format, to be readable from CC. For file format conversion, we used Global Mapper (GM) alongside CC. During manual registration, issue C occurred.

C. Elaborated manual registration required.

**Figure 5:**
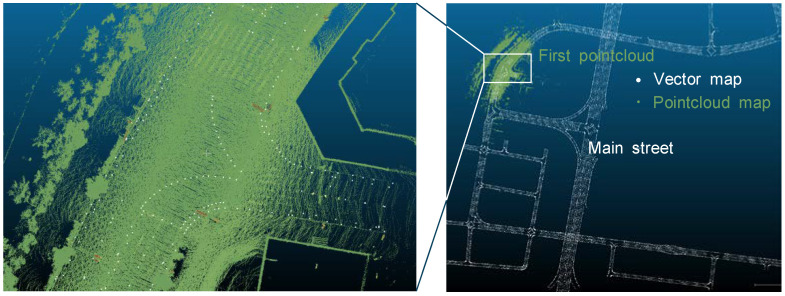
Difference in the number of points between the pointcloud and vector maps, which hindered the ICP.

It was difficult to accurately register such thin road marks, right from the beginning. To make it easier, we first roughly matched relatively large and apparent landmarks in the current pointcloud with those in the adjacent pointclouds. This helped to render the floating pointcloud closer to its goal position. Manual registration was completed using the iterative closest point (ICP)-based automatic registration offered by CC [41]. Despite the absence of a fixed criteria for registration accuracy, an RMS error less than 0.5 was considered acceptable. Automatic registration was specifically helpful in matching, along with the z-axis, which was much tougher to register manually than the other axes. However, we discovered that automatic registration could not always guarantee greater registration accuracy. This was because the ICP could not function properly, which was related to the large discrepancy between the two maps, in terms of the number of points. For example, a lane line was expressed by dozens of points in the pointcloud map; however, in the vector map, it was expressed by only two points. ICP iteratively minimized point-to-point distances between two pointclouds. For map registration, ICP selected the closest points as a correspondence, and calculated the transformation matrix between them. However, a much greater number of points in the pointcloud map could not find their corresponding points from the vector map (see Figure 5). Another challenge, which occurred in Step 3, was issue D.

D. Registered but inaccurate pointcloud, due to large coordinates.

**Figure 6:**
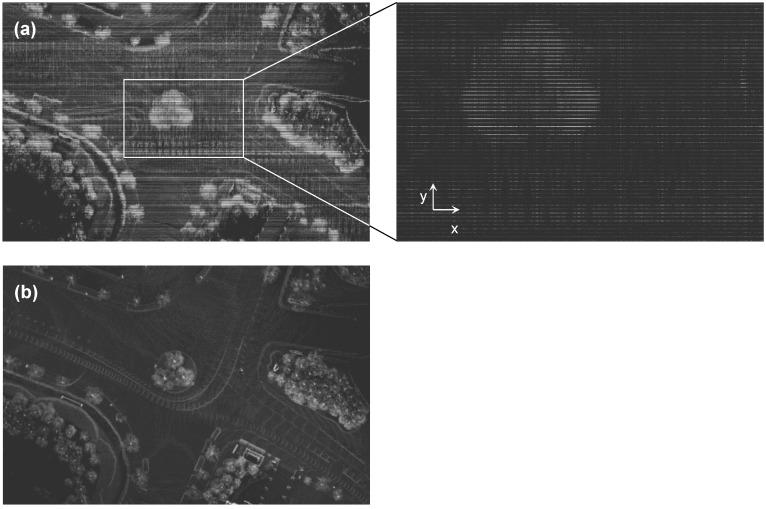
Comparison of the accuracy of pointcloud in (**a**) large coordinates (>10^5^ m) and (**b**) small coordinates (<10^3^ m).

In Step 3, the point coordinates were transformed relative to the global frame, based on the origin of the UTM52N zone. Here, the global frame was identical to the world frame. As a result, the magnitudes of the x and y coordinates were in the order of 10^5^ and 10^6^ m, respectively, which were denoted as large coordinates. For instance, the coordinates of a certain point in the transformed pointcloud at the contest site were 311,011 and 3,890,724 m, respectively. In addition to the coordinates of the transformed pointcloud, its format needed to be converted from TXT to PCD, using CC, which was compatible with Autoware. PCD is a file format designed to store 3D pointcloud data. The field that a point had was specified to the position in the 3D space (xyz) only. The color (rgb) was not necessary, because the road marks could be identified using the difference in brightness. CC is designed to reduce memory consumption by storing the point coordinates within 32-bits: namely, a single-precision floating-point format. This is usually acceptable for points that are a few kilometers (10^3^ m) apart from the frame origin, in which case the precision can be approximately a few micrometers (10^−6^ m). However, this is unacceptable for points that are more than 10^6^ m away. Importing such large coordinates within the 32-bits format is subject to a precision of several centimeters or worse. In consequence, we observed that the shape of the objects in the PCD-formatted pointcloud was misrepresented specifically along with the y-axis (see Figure 6). So far, the map frame was corresponding to the world frame. This meant that the map frame was relative to the origin of UTM52N zone; therefore, the transformed pointcloud relative to the map frame had such large coordinates. As a remedy for issue D, the map frame was moved nearby, while neglecting the world frame, which meant that the origin of the global frame was shifted closer to the center of the contest site; we named this as the map frame. The map frame minimized the magnitudes of the point coordinates. Consequently, the point coordinates could be stored within the 32-bits format without compromising the positioning accuracy. Accordingly, the vector map became relative to the map frame and, based on this, multiple local pointcloud maps were matched and transformed, as described above. Although prior map-based localization mostly depended on the LiDAR localizer, the GNSS localizer was also used to provide the initial position of the vehicle. Thus, the coordinates of the initial position were in the form of latitude, longitude, and height (llh). The LiDAR localizer started map-matching from the initial position provided by the GNSS localizer. These two localizers had to be based on the same frame. The GNSS localizer was modified to compensate for the coordinate transformation, by transforming the global frame into the map frame when converting the initial position from llh to xyz.

### 2.2. Map-Matching

We validated the registered pointcloud map, the result of map-building, by map-matching. Autoware provides two different types of ROS packages for map-matching: NDT_matching and ICP_matching. These two map registration algorithms, NDT and ICP, are compared in [42]. For prior map-based localization, the matching process needs to be performed in a 3D space populated with dense pointclouds. The scanned pointcloud is necessarily polluted by noise, owing to measurement errors, which are more serious outdoors. The pointcloud in the prior map is often ill-matched with the scanned pointcloud. This is possible if the concerned object actually changes after the map has been made. Otherwise, the concerned object is occluded from the LiDAR by another object. To make matters more demanding, the matching process needs to be performed in real-time. NDT_matching was selected to satisfy all these requirements. NDT_matching usually outperforms ICP_matching, in terms of computational efficiency and robustness.

**Figure 7:**
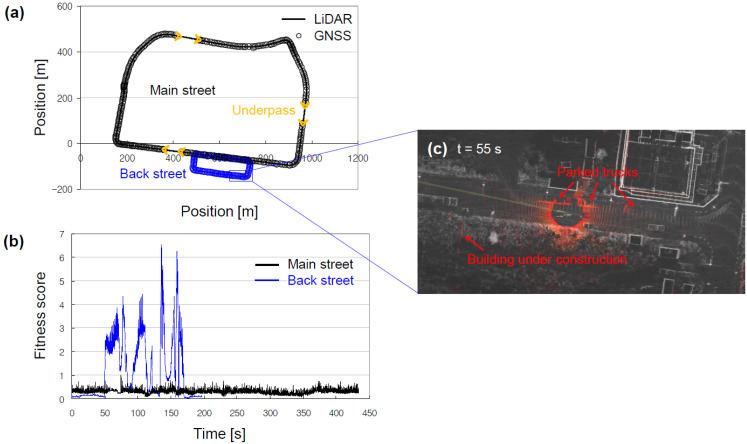
Validation results of the registered pointcloud map: (**a**) positions of the vehicle, measured by the LiDAR and GNSS localizers; (**b**) fitness score estimated from the LiDAR localizer; (**c**) fitness score negatively affected by unmapped objects.

The registered pointcloud map was evaluated using performance metrics that the NDT_matching package provided (see Figure 7). These included fitness score, execution time, and iteration number. It was found that the fitness score was maintained at a low level during map-matching on the main street. However, the fitness score surged when navigating through parked trucks or passing by a building under construction on the back streets. This was because such objects were scanned by the LiDAR but were not present on the prior map. The contest site was located within a new-town site, and construction work was in full swing at the time the contest was held. Such ill-matched pointclouds are a typical cause of NDT_matching failures. NDT_matching can also fail due to several other causes and their interplay, such as incorrect initialization, and limited computational resources. Regardless of the cause, localization failure undermines the overall reliability of AD, severely and instantly. To prevent unsafe situations, we designed the TOR to be issued without delay, if the performance metrics, mostly the fitness score, exceeded their thresholds.

## 3. Vehicle Control

Vehicle control refers to the task of enabling the ego-vehicle to follow a given path. A path in AD comprises an array of waypoints: namely, a discrete representation of the path. The path is provided by path-planning, which is configured by global, local, and behavioral planners. The global planner generates a path from the current location of the vehicle to the goal location. Accordingly, the local planner generates drivable and obstacle-free trajectories. The local planner is coupled with the behavior planner, to make a decision against diverse traffic situations in which the vehicle might operate while heading for the goal location. The generated trajectories are passed to vehicle control, so that they can be realized with the vehicle.

Vehicle control is configured with the waypoint follower and velocity controller. As an upper-level controller, the waypoint follower generates a velocity command that is a pair of linear and angular speeds. Among the relevant ROS packages that Autoware provides, pure_pursuit was selected. In every control cycle, pure_pursuit searches for the closest waypoint towards the heading of the vehicle, determining the next target waypoint. The target linear speed is predetermined by the local planner and, accordingly, the target angular speed is calculated with the radius of curvature. The generated velocity command is sent to a lower-level controller, which enables the vehicle to reach the next target waypoint within the next control cycle. The velocity controller generates a set of throttle, brake, and steering commands to achieve the velocity command to the vehicle.

Accordingly, we employed the third-party ROS package, which implemented the velocity controller with two separate proportional–integral–derivative (PID) controllers, for longitudinal and lateral speeds [43]. The longitudinal speed controller took two inputs: namely, the velocity command from the waypoint follower, and the speed measurement feedback from the controller area network (CAN) bus of the vehicle. The error between the reference and actual speeds was minimized with the tuned PI gains, and accordingly generated the throttle and brake commands in pedal depth (BPS). The lateral speed controller also received the velocity command and steering wheel angle measurement feedback. Based on a simple kinematic bicycle model, the angular speed in the velocity command was converted to an angle. Similarly, the error between the reference and actual angles generated the steering command in torque.

### 3.1. Problem Statement

**Figure 8:**
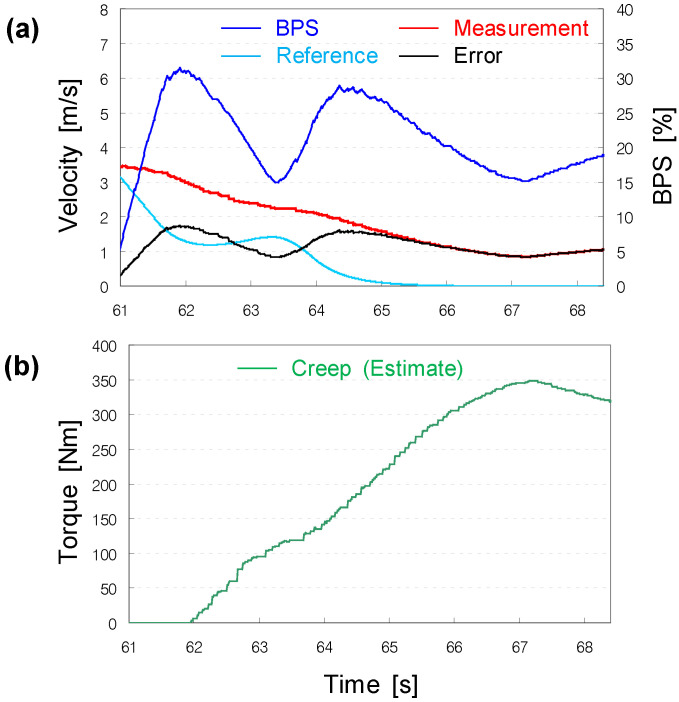
Problem of the PID-based longitudinal speed controller: (**a**) despite a zero-velocity command from 65 s, the vehicle did not stop, presumably due to (**b**) creep torque.

We observed that the PID-based longitudinal speed controller was good at tracking the velocity command at intermediate speeds; however, with a zero-velocity command, it could hardly stop the vehicle (see Figure 8). It was alleged that this problem was mainly derived from creep torque. The creep torque is part of the traction torque produced by the powertrain with an automatic transmission, such that a vehicle can creep forward. While the traction torque is determined by the longitudinal speed controller, the creep torque is produced irrespective of the throttle command. The creep torque is generated only if the vehicle stops or moves at low speeds. Normally, the creep torque is maximized at near-zero speeds, and then tapers off as it approaches a specific speed. However, the creep torque profile differs from model to model. The problem we encountered was most likely to have occurred because the traction torque generated to stop the vehicle was disturbed by the creep torque applied to move it forward. The creep torque could be considered as external disturbance, since it was not completely accounted for by the vehicle model implemented in the longitudinal speed controller. In addition, external disturbance included changes in the gross weight of the vehicle and the slope of the road.

In this section, we present a procedure for developing the robust longitudinal speed controller based on a disturbance observer (DOB). DOB-based control algorithms first appeared in the late 1980s; since then, they have been applied to many applications, and have achieved good tracking performance under external disturbance and model uncertainty [44]. DOB-based control algorithms have also been applied to vehicle speed control, and have achieved good disturbance-rejection performance [45].

### 3.2. DOB

The developed procedure consisted of vehicle model derivation, DOB-based controller design, validation by computer simulations, integration, and validation by physical tests.

#### 3.2.1. Vehicle Model Derivation

First, we defined a longitudinal vehicle model, and experimentally identified its parameters, which served as a nominal plant model in the DOB-based controller. The model described the forces acting on the vehicle in the longitudinal direction. These forces could be grouped into the tire force (forward) and resistance force (backward). The resistance force was the sum of the rolling $F_{roll}$, air $F_{air}$, and gradient $F_{grad}$ resistances, if a road was inclined. The tire force $F_{tire}$ was the traction torque generated by the powertrain. Based on Newton’s second law, the imbalance between these forces yielded the longitudinal vehicle acceleration:

(1) $$ma=F_{tire}-F_{roll}-F_{air}-F_{grad}$$

Equation (1) represents the plant model that described the longitudinal vehicle motion; however, it was difficult to completely characterize these forces, because of external disturbance and model uncertainty. For instance, the resistance force changed with the gross weight of the vehicle and the slope of the road. As observed with the PID-based controller, the tire force was changed by the creep torque applied at low speeds.

The current controller was reinforced by the DOB as a corrective measure to prevent or reduce such undesirable effects. The DOB is designed to lump external disturbance and model uncertainty collectively, and to compensate for them in the feedback loop. Accordingly, the vehicle model in Equation (1) was redefined with a lumped mass, resulting in the nominal plant model:

(2) $$m_{n}a=F_{tire}$$

The first-order system in Equation (2) can be rewritten in the form of a transfer function as:

(3) $$P_{n}\left(s\right)=\frac{v\left(s\right)}{F_{tire}\left(s\right)}=\frac{1}{m_{n}s}\;$$

The lumped mass of vehicle $m_{n}$ in Equation (2), which involved all the resistance forces, was experimentally extracted from the result of the vehicle acceleration test; $F_{tire}$ was directly calculated from the motor torque measurement, combined gear ratio, and effective tire radius; a was estimated from the vehicle speed measurement, which required signal processing to filter out noise. Consequently, $m_{n}$ was determined to be 1540 kg. As mentioned previously, $m_{n}$ was obtained under specific conditions. Therefore, it was not generally applicable—for example, at up/down-hill, with more payloads, and at low speeds where the creep torque arose. Nevertheless, the DOB could function with $m_{n}$ under a wide range of conditions; therefore, computationally expensive plant models were not required.

#### 3.2.2. DOB-Based Controller Design

**Figure 9:**
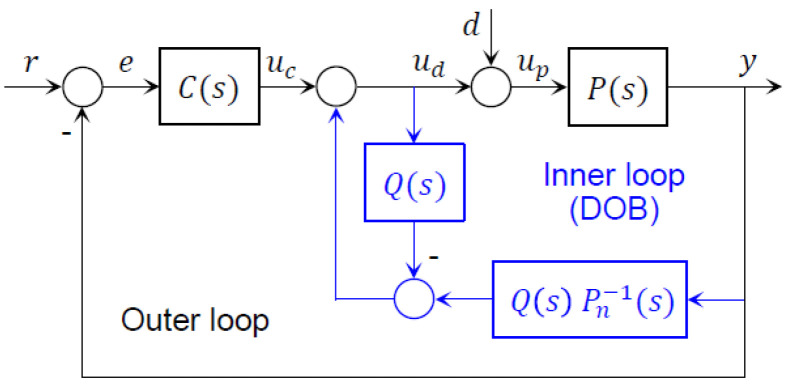
Block diagram of a longitudinal speed controller with DOB.

Subsequently, we developed the DOB-based controller, by reversing the derived nominal plant model to compensate for the disturbance. The structure of the DOB-based controller was composed of two nested loops (see Figure 9). While the outer loop involved a conventional PI controller, the inner loop involved a DOB.

Within the inner loop, $P_{n}\left(s\right)$ represented the nominal plant model of P(s); $P_{n}^{-1}\left(s\right)$ indicated the inverse nominal plant model, that reversed the input and output of $P_{n}\left(s\right)$; Q(s) denoted the Q-filter, which was considered as a filter, because it was in the form of a low-pass filter:

(4) $$Q\left(s\right)=\frac{a_{0}}{{\left(\tau s\right)}^{l}+a_{l-1}{\left(\tau s\right)}^{l-1}+\cdots +a_{1}\left(\tau s\right)+a_{0}}$$

where τ was the time constant, and l was the relative order. In Equation (4), l was properly selected, such that $Q\left(s\right)P_{n}^{-1}\left(s\right)$ could be a proper transfer function, in which the degree of the numerator did not exceed the degree of the denominator. Hence, l was set to 1, as $P_{n}\left(s\right)$ was the first-order system. It was necessary to carefully determine τ, by considering the complementary characteristics of the DOB. In principle, τ was inversely proportional to the bandwidth of Q(s). Therefore, the smaller τ was, the higher the disturbance-rejection performance, yet the smaller the sensor noise immunity the DOB obtained. In practice, τ was selected between the sampling time and the natural frequency of the system. While the lower limit was simply determined by the control cycle, the upper limit was typically unclear. Therefore, it was safer to set τ as the near-minimum value that could satisfy the lower limit, which was fixed at 0.1.

Within the outer loop, P(s) represented the actual plant model with uncertainty; $u_{p}$ and y denoted the input and output, respectively; C(s) represented the controller model that minimized the error between y and the reference input, r. C(s) is designed for $P_{n}\left(s\right)$, meaning that it did not consider external disturbance and model uncertainty. Therefore, the DOB within the inner loop was designed in such a way that the system, except C(s), could behave like $P_{n}\left(s\right)$. With the DOB, the system with C(s) could operate as expected.

#### 3.2.3. Validation by Computer Simulations

We then validated the robustness of the DOB-based controller, using Simulink. The structure of the DOB-based controller contained two subsystems: controller and vehicle: the controller output the torque demand in response to the inputs of the reference and actual speeds; the vehicle took the torque demand as an input, and output the actual speed. As another input to the vehicle, the torque and weight variations were added, to simulate external disturbance and model uncertainty, respectively.

In the vehicle subsystem, the actual plant, P(s), in the outer loop was modeled as a first-order system. In this model, the equivalent rotational inertia of the vehicle was calculated at the wheels (on the wheel-side):

(5) $$J=mr^{2}$$

where m was the vehicle weight, and r was the wheel radius. As required by the controller, the torque demand, T, acting on the vehicle with J, caused the rotational acceleration of the wheel, α. Integrating α, with respect to time, gave their rotational velocity, ω. Multiplying ω by the wheel radius, r, finally yielded the linear velocity of the vehicle, v. As described above, the longitudinal vehicle motion could be simply modeled; however, in reality, J was subjected to model uncertainty, while T was susceptible to external disturbance.

By passing v through Q-filter Q(s) in the inner loop, the actual speed was then fed back to the controller subsystem. The controller subsystem primarily consisted of the P controller and DOB blocks. In addition, two rate-limiter blocks were applied to the reference speed and torque demand. The rate-limiter limited the first derivative of the input, such that the output could change no faster than the specified limit. In the P controller block, the controller C(s) in the outer loop was implemented, which generated the torque demand. In the DOB block, the nominal plant, $P_{n}\left(s\right)$, is inversely modeled, in that the process in the vehicle subsystem was reversed. The only difference was that the equivalent rotational inertia of the vehicle was calculated for the electric motor (on the motor-side):

(6) $$J=\frac{mr^{2}}{G^{2}}$$

where G was the gear ratio between the rotational velocities of the wheel and that of the motor; subtracting it from T produced the observed disturbance, $\hat{d}$. By passing $\hat{d}$ through Q(s) in the inner loop, another torque demand was generated, to compensate for the disturbance. This was added to the torque demand generated by the P controller block, finally yielding the resultant torque demand. To verify the robustness to the external disturbance, a function defined by the creep torque profile was added to the resultant torque demand.

**Figure 10:**
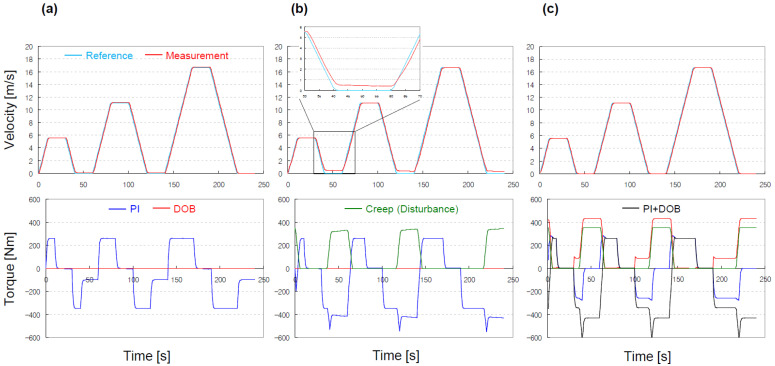
Validation results of the DOB-based controller: (**a**) without creep torque; (**b**) with creep torque as external disturbance, the vehicle never stopped, as observed in Figure 8; (**c**) with P+DOB controller, the vehicle could stop, despite creep torque.

In the simulation, ramp and constant functions were used to create the reference speed profile. While tracking the reference speed profile, the DOB-based controller (P + DOB controller) was compared to the PI controller, in terms of disturbance rejection performance. The relevant parameters in each controller were tuned in advance, to show equivalent tracking performance against the reference speed profile (see Table 4). We observed that the two controllers exhibited an equivalent level of tracking performance at intermediate speeds (see Figure 10). However, compared with the P + DOB controller, the PI controller exhibited a larger tracking error at low speeds where the creep torque increased. This is primarily because the integral term in the PI controller was not appropriate for effectively reducing the external disturbance.

#### 3.2.4. Integration

Instead of hand-writing the code, we used Embedded Coder to auto-generate C++ code from the Simulink model of the controller subsystem in the DOB-based controller. It should be noted that the Simulink model of the vehicle subsystem was not subjected to code generation, because the generated code was used with a physical vehicle. Embedded Coder extended MATLAB Coder and Simulink Coder with advanced optimizations; for instance, the generated code was compact, and executed efficiently, which is essential for real-time computing in embedded systems.

The generated code included the main, model, data, and utility files. The main file (ert_main.cpp) controlled code execution by calling the entry-point function to step the model for a base rate. The model file implemented the DOB-based controller in C++, and its header file declared the model data structures and a public interface to the model entry points. The data file contained model parameters to be calibrated. The utility file defined the data types, structures, and macros required for the generated code. Owing to Embedded Coder, we did not have to write the code; however, we were required to write an ROS wrapper for the generated code, so that it could function with the associated ROS packages, such as pure_pursuit. In the wrapping process, the header file that declared the interface to the model was modified, to create a new ROS package called twist_controller. The detailed process is introduced elsewhere, because it is not in line with the purpose of this section.

#### 3.2.5. Validation by Physical Tests

**Figure 11:**
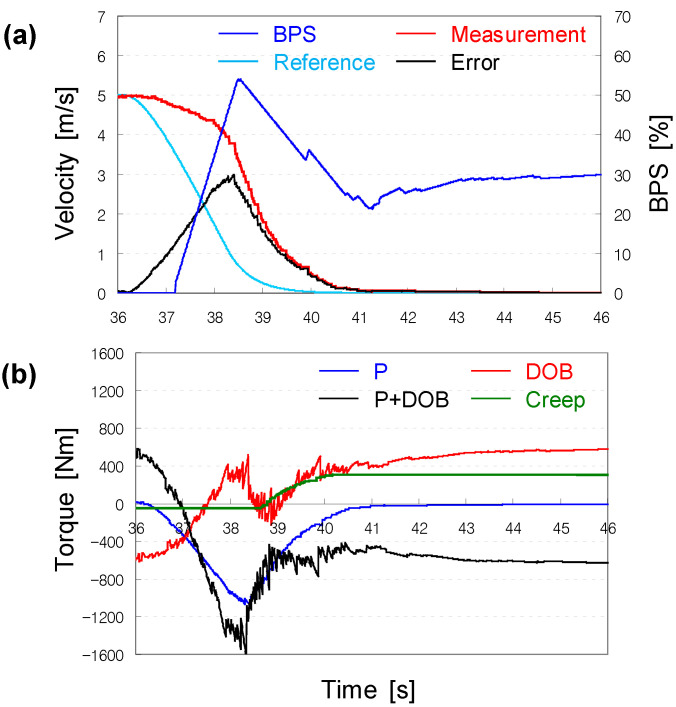
Validation results of the DOB-based controller: (**a**) contrary to Figure 8, the vehicle could stop; (**b**) with P + DOB controller, despite creep torque.

As observed in the prior computer simulations, the P + DOB controller maintained a small tracking error over the entire speed range, because the DOB could estimate and compensate for the external disturbance effectively (see Figure 11).

## 4. V2I Communication

V2I communication refers to the task of wireless data exchange between the RSU and vehicles. With the data being obtained and shared, rich, timely, and NLOS-aware traffic information can be utilized to enable a variety of benefits in AD.

Currently, two different wireless communication technologies exist: dedicated short-range communication (DSRC) and cellular-V2X (C-V2X). A detailed comparison between these two standards can be found in [46]. Essentially, both standards have almost the same use cases in real-life applications. Whatever both sides claim, there are, as yet, no side-by-side test results that prove that one outperforms the other. Initially, C-V2X seems to offer greater performance; however, the range and latency of DSRC is more than sufficient for the purpose of AD. Although C-V2X exhibits better long-term prospects, owing to a lack of statistical evidence, related industries are faced with a difficult choice as to which one to adopt in their communication infrastructure.

Reflecting such a transitional situation, both technologies were concurrently applied in the contest for different purposes. While information related to the robo-taxi service—such as the pick-up and drop-off locations—was broadcast with C-V2X, information related to a signalized intersection, at which vehicles in the contest might cross while heading toward these goal locations, was broadcast by DSRC. In this section, we describe a procedure for applying V2I technology to the TLR and a robo-taxi service handler (TSH).

### 4.1. TLR

**Figure 12:**
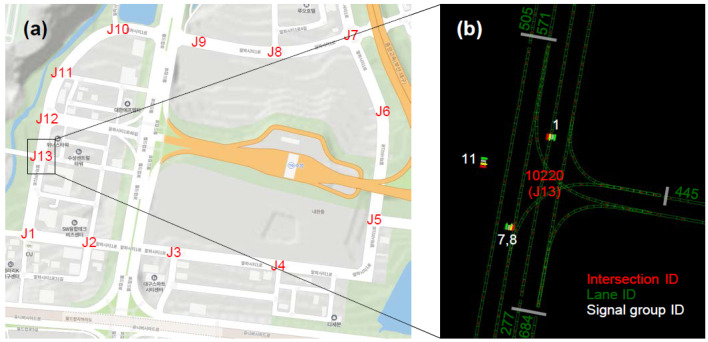
(**a**) Locations of 13 signalized intersections in the contest site: (**b**) 13th intersection (J13), represented with the intersection, lane, and signal group IDs.

There were 13 signalized intersections all over the contest site; therefore, the vehicles were likely to cross them on the way to their goal locations (see Figure 12). At the intersection, the vehicles had to obey traffic rules, or else earned points were deducted. As mentioned previously, if the violated traffic rule was a traffic signal, 10 times more points were lost. A camera that was used for the TLR was substituted for the V2I communication system, to achieve a nearly perfect recognition rate.

**Figure 13:**
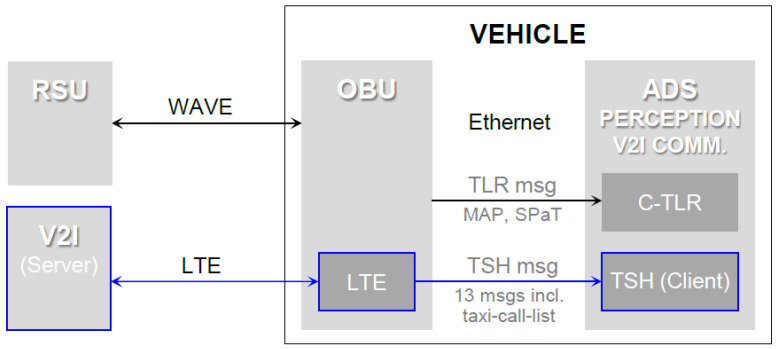
High-level physical representation of the V2I communication interface.

Figure 13 shows a high-level physical representation of the V2I communication interface. For traffic light recognition, packets were exchanged between the RSU at the intersection and the on-board unit (OBU) in the vehicle, through DSRC, also known as wireless access in vehicular environments (WAVE). The TLR packets were composed of header and payload. The header contained information regarding the message type, device (OBU) type, ID, and payload length. While the header length was fixed at nine bytes, the payload length varied, depending on the amount of content in the message. The TLR messages in the payload were decoded by UPER, to extract traffic signal information in the J2735 format. The WAVE broadcasts of the SPaT message conveyed the real-time signal phase and timing status for one or more intersections, whereas the MAP message transmitted the complex intersection geometry required to decode the SPaT message. A new ROS package, named V2I communication-enabled TLR (C-TLR), was created and added to the perception module.

#### 4.1.1. Tasks

The C-TLR was required to decode the MAP message, to select a signal head that the ego-vehicle had to follow. The C-TLR was also required to decode the SPaT message, to extract the current phase and its remaining time from the selected signal head.

#### 4.1.2. Inputs and Outputs

The C-TLR interfaced externally with the OBU, to receive the MAP and SPaT messages from the RSU via WAVE, in order to accomplish the two tasks mentioned above. In the MAP message, the intersection ID that the vehicle approached was taken as an input. In the SPaT message, the real-time signal phase and timing status of the intersection were accepted. The C-TLR interfaced internally with the local planner, to take the lane ID in which the vehicle was located as another input. With the inputs of the intersection ID and lane ID, the C-TLR internally output the signal group ID. The C-TLR output the current phase of the signal group ID to the behavior planner, to transition the state of the vehicle crossing the intersection accordingly; for instance, from FORWARD to STOPPING, TRAFFIC_LIGHT_STOP, and TRAFFIC_LIGHT_WAIT.

#### 4.1.3. Process

The process of the C-TLR can be summarized as selecting a signal head to follow, and providing the signal phase and timing status to the behavior planner. A typical intersection had a set of connected lanes, and each lane had a signal head that the vehicle in that lane should follow. Thus, the current lane ID was required to select the signal head for the vehicle. Originally, the lane ID had been identified by matching the location of the vehicle with that of each lane. The location of each lane was provided with the MAP message with respect to llh. The location of the vehicle had to provide lane-level accuracy. For this, a GNSS localizer needed a clear LOS to the satellites; however, if the intersection was located at urban environments, it was difficult to ensure the actual positioning error levels. It was not necessary to obtain the lane ID from the MAP message with such difficulty, using the vector map. With the lane ID published by the local planner, and the intersection ID decoded from the MAP message, the signal group ID could be readily retrieved from the predetermined table in the C-TLR (see Table 5 and Figure 12b). The current phase of the selected signal group ID was then published to the behavior planner. Using its end time, the phase was recalculated with the distance to the stop line and the velocity of the vehicle. For instance, although the current phase was blue, the C-TLR could be set to red, if the vehicle could not safely pass the conflict area of the intersection within the remaining time at the current velocity.

### 4.2. TSH

V2I technology was not necessary for traffic light recognition, because the vision-based TLR was still useful; however, it was essential for the TSH because, in the contest, virtual calls for a ride were broadcast over the air.

As shown in Figure 12, the OBU that had been used for the TLR was also used for the TSH. The TSH acted as a client, and connected to the server via LTE. The TSH packets were also composed of header and payload, but the messages within the payload were different to those in the TLR packets. While the TLR messages, such as MAP and SPaT, were general and standardized, the TSH messages were prepared for this contest only. As listed in Table 6, 13 TSH messages were introduced by presenting the problem scenario.

#### 4.2.1. Problem Scenario

The problem scenario of the robo-taxi service consisted of the following seven steps:

1. The server broadcast a ‘taxi-call-list’ message every second;
2. The client (TSH) had to select one desired mission from the received call list;
3. Once a mission had been selected, the vehicle sent a ‘pre-empt call’ message to the server;
4. The server checked if the selected mission was currently available, and sent a ‘call-matched’ message to the vehicle;
5. If the selected call was matched, the vehicle departed to pick up virtual passengers. At the starting point of the mission, the vehicle sent an ‘arrive-at-start-point’ message to the server, and stopped for five seconds. The on-site supervisors directly checked the behavior of the vehicle, and sent an ‘arrive-at-start-point-checked’ message to the server through their mobile terminal. If successful, the server sent a ‘pick-up-completed’ message to the vehicle. If not, a ‘mission-failed’ message was sent instead. The vehicle had to revert to Step 2, and restart another mission. However, as a penalty, the next mission could not be pre-empted for another 10 seconds;
6. Once the ‘pick-up-completed’ message had been received, the vehicle departed again, to drop off virtual passengers. At the end-point of the mission, the vehicle transmitted an ‘arrive-at-end-point’ message to the server, and stopped for five seconds. In the same manner, the supervisors sent an ‘arrive-at-end-point-checked’ message to the server. If successful, the server sent a ‘drop-off-completed’ message to the vehicle; otherwise, the ‘mission-failed’ message was sent. As in the previous step, the vehicle had to revert to Step 2, and resume with the penalty.
7. Once the ‘drop-off-completed’ message was received, the vehicle had to depart from the destination within a minute. The supervisors sent a ‘depart-from-end-point-checked’ message to the server. If successful, the server sent a ‘mission-completed’ message to the vehicle. Finally, the team acquired the points awarded for the completed mission, and the next mission could be pre-empted instantly in Step 2. If not, a ‘mission-failed’ message was sent. As in the previous step, the vehicle had to revert to Step 2, and resume with the penalty.

#### 4.2.2. Tasks

The TSH was required to select the optimal call by comparing 47 calls broadcast from the server. The TSH had external access to the server to receive the call list. The TSH internally communicated with the global planner, to evaluate the cost of traversal through the path within each call. The TSH was also required to decode the matched call, particularly in terms of the locations of the pick-up, drop-off, and possible lane closure between them. As these locations were provided in the form of llh, the TSH transformed them into the map frame to communicate with the global planner. The global planner generated the path based on the map frame where these locations were expressed as xyz, with respect to the origin that was moved into the contest site.

#### 4.2.3. Inputs and Outputs

The TSH interfaced externally with the server via LTE, and internally with the global planner, to perform the two tasks mentioned above. For selecting a taxi call, the TSH first took a ‘taxi-call-list’ message, as input from the server. As mentioned previously, the call list consisted of 47 calls, each of which primarily contained the locations of the pick-up, drop-off, and possible lane closure between them. These locations needed to be transformed from llh to xyz. The TSH internally output the transformed locations, to calculate the cost of traversal through the path in each call from the global planner. The TSH took the calculated cost as feedback, and the cost function yielded the final cost. The cost function used two more factors than the cost that the global planner provided, which were the point and distance. The call with the lowest final cost was determined to be the mission to be pre-empted. The TSH accordingly output a ‘pre-empt-call’ message to the server. The TSH then took a ‘call-matched’ message as input, and decoded the matched call. As mentioned previously, the transformed locations were represented as a set of points (xyz) in the map frame. The pick-up and drop-off locations were defined by a single point, whereas the lane closure location was defined by multiple points, thus, a plane. If the lane closure location was included in the matched call, the number of its points was augmented, to help the global planner generate a path that could avoid it more safely and efficiently. The TSH output the processed locations to the global planner. When the vehicle reached the pick-up or drop-off location, the TSH took an arrival notification as input from the local planner, and relayed it with an ‘arrive-at-start’ or ‘end-point’ message to the server. After being checked by the supervisor, the TSH finally received a ‘pick-up’ or ‘drop-off-completed’ message as input from the server.

#### 4.2.4. Process

**Figure 14:**
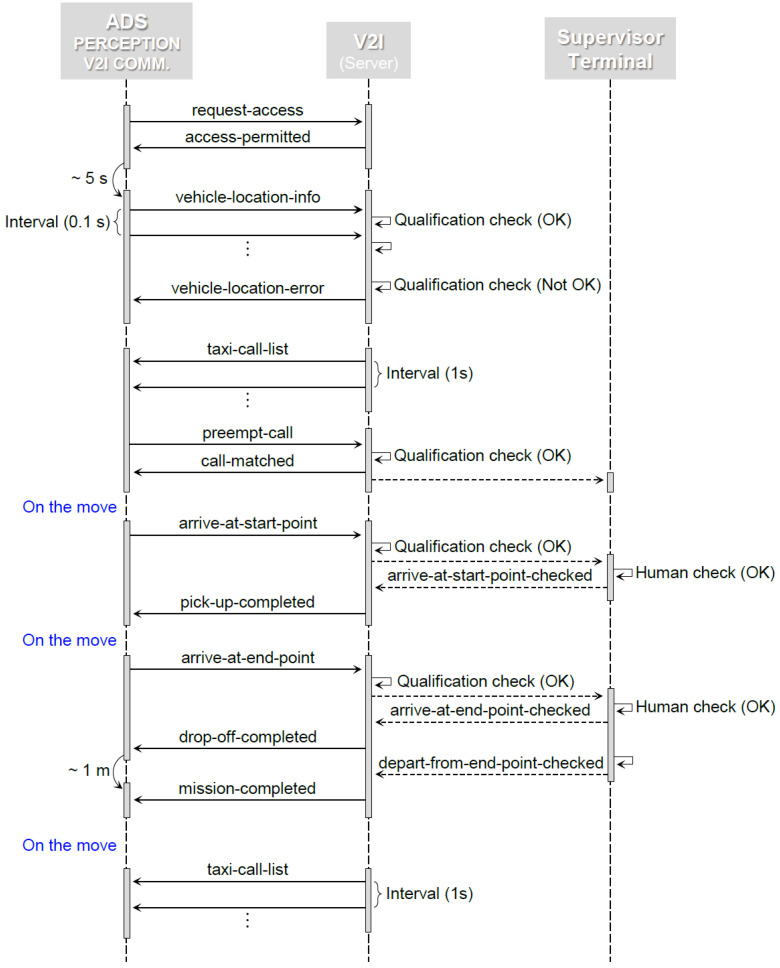
Process of the TSH, represented by a sequence diagram.

The process of the TSH was implemented primarily using a finite state machine (FSM). The TSH comprised eight states, and the active state was determined based on 13 input conditions that yielded a logical output. If the starting state was ‘request-connection’ state, the active state was instantly shifted to the ‘get-connection-confirmed’ state, by sending a ‘request-access’ message to the server. If an ‘access-permitted’ message was sent back, the active state was changed to the ‘select-call’ state; otherwise, the active state was shifted back to the ‘request-connection’ state. In the select-call state, one desired mission was selected out of 47 calls broadcast over vehicles in the contest. As described previously, each call was evaluated based on a cost function with three different factors: point, distance, and cost. The first and second factors were given by the server. The point varied, depending on the mission’s degree of difficulty. In addition to the distance, left turns, tunnels, areas not on the HD map, and lane closure between the pick-up and drop-off locations determined the point. The distance given in the call was simply between the pick-up and drop-off locations. However, the distance from the current location to the pick-up location also needed to be considered. Moreover, the final cost could not be determined simply by the physical displacement between the locations. For these reasons, the third factor was added, and calculated using the global planner. The three factors were properly weighted, yielding the final cost. The final costs of the 47 calls were sorted in descending order, and the call with the lowest final cost was determined as the mission to be pre-empted. The active state was shifted to the ‘get-call-confirmed’ state, by transmitting a ‘pre-empt-call’ message to the server. If a ‘call-matched’ message was responded to, the active state was changed to the ‘pick-up-passenger’ state, where the vehicle departed to the starting point of the mission. If the desired call was already pre-empted, for instance, the active state reverted to the ‘select-call’ state, to pre-empt the call with the second-lowest final cost. When the vehicle reached the pick-up location, the active state shifted to the ‘get-arrival-confirmed’ state, and an ‘arrive-at-start-point’ message was sent to the server. The location of the vehicle was double-checked by the on-site supervisors, and an ‘arrive-at-start-point-checked’ message was subsequently sent to the server through their mobile terminal. If a ‘pick-up-completed’ message was sent back, it progressed the active state to the ‘drop-off-passenger’ state, where the vehicle departed to the end-point of the mission. If not, it returned the active state to the ‘pre-empt-call’ state, to restart another mission. When the vehicle reached the drop-off location, similar to the ‘pick-up-passenger’ state, the active state shifted to the ‘get-arrival-confirmed’ state, and an ‘arrive-at-end-point’ message was sent. In the same fashion, the location of the vehicle was double-checked by the supervisors. If a ‘drop-off-completed’ message was received, it progressed the active state to the ‘mission-completed’ state. In the final state, the supervisors checked if the vehicle had departed from the end-point of the mission within one minute, and subsequently sent a ‘depart-from-end-point-checked’ message to the server. If a ‘mission-completed’ message was acquired, the started mission was considered completed. The active state then regressed to the ‘select-call’ state, to continue another mission. The described process is illustrated, using a sequence diagram to show the interactions between objects in the sequential order (see Figure 14).

## 5. Discussion

We entered the 2020 University Student Autonomous Driving Contest. The main mission was to transport passengers to their destination autonomously: in short, to implement a robo-taxi service on public roads. In this paper, we have presented the AD software stack developed to complete the main mission of the contest. Among the major developments required for the main mission, this paper focused on HD mapping, vehicle control, and V2I communication, which were not supported by Autoware. The methods we applied to each of the developments are summarized in Table 7, with emphasis on their benefits.

## 6. Conclusions and Future Work

Thanks to our successful developments, we finished 5^th^ out of 15 teams in our first contest. We would have done better, if there had been no problem with the vehicle. About an hour after the start of the contest, the vehicle suddenly stopped, with an unidentified alarm. We suspected that DriveKit, which acts as an integrated vehicle interface for full by-wire control (see Table 2), was the cause of the problem, because removing it from the vehicle solved the problem. DriveKit is a finished product, and thus it was not possible to take immediate action. With this problem in mind, we have been designing our own actuator, expecting free maintenance and possible upgrades for our specific needs.

Another problem we will address is related to the vehicle speed. Although the speed limit in the contest site is 50 km/h, we could only drive up to 35 km/h on straight roads and 20 km/h on curved roads. We have been applying a model predictive algorithm into a waypoint follower to increase the maximum speed, since the current pure pursuit algorithm is limited to low-speed maneuvers only. In addition, we need to analyze the end-to-end latency over the AD software stack, to ensure a responsiveness that is as fast as the increased speed.

## Figures and Tables

**Table 1:** Factors and their weights applied to calculate the points in the contest.

Points	Factors	Weights
earned by	Left turns	+0.1
	Underpasses	+0.2
	Roads off the map	+0.4
	Lanes closed for construction	+0.5
deducted by	All traffic law violation (except traffic signal)	−0.1
	Traffic signal violation	−1
	TOR	−0.3

**Table 2:** Hardware specifications of the AD system and its base vehicle.

		Model	Specifications
Base vehicle		Kia SOUL EV 2018	30 kWh battery, 81.4 kW/285 Nm electric motor
Sensors	LiDAR	Ouster OS1-64	64 channel, 120 m range, 3 cm accuracy
	Camera	FLIR Grasshopper3 GS3-U3-32S4C-C	Color, 3.2 MP, 121 FPS at 2048 × 1536
	GNSS	JAVAD Delta-3	1 cm (horizontal), 1.5 cm (vertical) RTK accuracy
	OBU	VW400-OBU-BK	WAVE/LTE/GPS modules, CAN/Ethernet/Console/USB ports
Computers		ZOTAC MAGNUS EK71080 ASRock DeskMINI 310	i7-7700, GTX 1080, 32GB RAM, 512 GB SSD i9-9900, 32GB RAM, 2TB SSD
Vehicle control interface		PolySync DriveKit	Throttle/Brake/Steering controls

**Table 3:** List of issues that occurred during map-building. All the issues were solved in Step 3: Registration.

Issues		Occurred in Step
A	Pointclouds scanned, relative to the local frame.	1, 2
B	Pointclouds ill-matched by approximate_NDT_mapping package.	2
C	Elaborated manual registration required.	3
D	Registered but inaccurate pointcloud, due to large coordinates.	3

**Table 4:** Predetermined parameters of the controllers.

Controllers	Parameters	Value
PI	P-gain	200
	I-gain	10
P + DOB	P-gain	200
	Time constant of Q-filter (*τ*)	0.1

**Table 5:** Signal group ID as a function of the intersection (from SPaT message) and lane (from vector map) IDs. For lack of space, only three (J2, 12, and 13) out of 13 signalized intersections are presented. The 13th intersection (J13) is in agreement with Figure 12b.

**Input**	**Intersection ID**	10220 (J13)					10210 (J12)				10020 (J2)					…
	**Lane ID**	445	505	571	277	684	654	655	662	536	484	173	158	355	544	
**Output**	**Signal group ID**	11	7	8	1	1	7	7	1	1	4	5	8	10	10	

**Table 6:** TSH messages and their key data.

Messages	Key Data
request-access	deviceId
access-permitted	responseStatus, errorCode
vehicle-location-info	latitude, longitude, speed
vehicle-location-error	errorCode
taxi-call-list	callDataList, irregularLocationList (i.e., the location of the lane closure)
pre-empt-call	requestCallId
call-matched	responseStatus, errorCode
arrive-at-start-point	latitude, longitude, speed
pick-up-completed	confirmResult, errorCode
arrive-at-end-point	latitude, longitude, speed
drop-off-completed	confirmResult, errorCode
mission-completed	confirmResult
mission-abandoned	matchingCallId

**Table 7:** Comparison between the conventional and proposed methods.

	Conventional Methods	Proposed Methods	Key Benefits
HD Mapping	Dedicated MMS	In-house developed	Can generate the HD (pointcloud) map at no extra cost, using a LiDAR on the vehicle, and open-source software.
Vehicle Control	PI	P + DOB	Can stop the vehicle smoothly, by rejecting undesired torque variations at low speeds (creep torque).
TLR	Vision	V2I communication	Can achieve nearly perfect recognition rate, due to NLOS awareness.
